# S-1 plus apatinib as first-line palliative treatment for stage IVB gastroesophageal junction adenocarcinoma: A case report and review of the literature

**DOI:** 10.1097/MD.0000000000018691

**Published:** 2020-01-03

**Authors:** Chu Zhang, Guang-Mao Yu, Miao Zhang, Dong Liu

**Affiliations:** a Department of Thoracic Surgery, Shaoxing People's Hospital (Shaoxing Hospital, Zhejiang University School of Medicine), Shaoxing; b Department of Thoracic Surgery, Xuzhou Central Hospital Affiliated to Southeast University, Xuzhou, China.

**Keywords:** angiogenesis inhibitor, apatinib, chemotherapy, S-1, targeted therapy, vascular endothelial growth factor receptor

## Abstract

**Rationale:**

Apatinib has been proven to significantly prolong the survival of the patients with advanced chemotherapy-refractory gastric cancer. To date, studies on apatinib plus S-1 as first-line palliative therapy for metastatic gastroesophageal junction (GEJ) cancer are rare.

**Patient concerns:**

A 61-year-old female patient was admitted with dysphagia, significant loss of body weight, and poor performance status.

**Diagnoses:**

Endoscopic biopsy revealed the diagnosis of poorly-differentiated GEJ adenocarcinoma, and the patient was clinically staged as T3NxM1G3 (IVB).

**Interventions:**

She had received 4 cycles of palliative therapy using oral apatinib (425 mg daily) plus S-1 (40 mg twice daily for 4 weeks, with a 2-week drug-free interval), followed by maintenance low-dose apatinib (250 mg daily) plus S-1 at the same dosage thereafter.

**Outcomes:**

Her progression-free survival was nearly 5 months, and the overall survival was >11 months up to now. The adverse events were tolerable.

**Lessons:**

Apatinib plus S-1 might be an alternative option for late-stage GEJ cancer. However, high-quality trials are warranted before the recommendation of this therapeutic regimen.

## 1 Introduction

Gastric cancer is the fifth most common malignancy and the third leading cause of cancer mortality worldwide,^[1]^ and the majority of the patients are diagnosed as advanced disease on admission. Palliative therapy in addition to best supportive care for stage IV gastric or esophageal cancer patients with poor performance status is aimed at providing a survival benefit or improved quality of life, however, the optimal first-line therapeutic regimen remains controversial.

Apatinib, a small-molecule tyrosine kinase inhibitor targeting vascular endothelial growth factor receptor-2 (VEGFR-2), could be considered as a third-line option for refractory gastric or gastroesophageal junction (GEJ) cancer.^[2]^ To date, the evidence regarding the combined use of apatinib and S-1 as first-line treatment for metastatic GEJ adenocarcinoma is insufficient. Herein we present a case of late-stage, poorly-differentiated GEJ adenocarcinoma in which the diffused liver metastases shrank and maintained stable for half a year since the administration of S-1 plus apatinib.

## 2 Case presentation

The clinical data of this patient was treated anonymously for privacy concern. A 61-year-old woman was admitted on January 21, 2019 because of abdominal pain and black stool since November 2018, after experiencing dysphagia and weight loss of nearly 10 kg over the preceding 6 months. Her previous medical history was unremarkable. Physical examination showed emaciation and a noticeably enlarged abdomen, with a body mass index (BMI) of 19.0. Besides, laboratory tests indicated mainly normal blood cell counts, renal function, neuron-specific enolase (NSE), carcinoembryonic antigen (CEA), carbohydrate antigen (CA) 724, and cytokeratin-19 fragment (CYFRA 21-1) but decreased serum hematocrit (19.3%, normal range 35–45%), hemoglobin (53 g/L, normal range 110–150 g/L), and albumin (31.5 g/L, normal range 35–55 g/L), followed by elevated CA125 (629.2 U/mL, normal range 0–35 U/mL). Additionally, impaired hepatic function as indicated by elevated serum aspartate aminotransferase (AST) (175.0 U/L, normal range 1–40 U/L), alanine aminotransferase (ALT) (56.0 U/L, normal range 1–40 U/L), alkaline phosphatase (ALP) (248 U/L, normal range 15–140 U/L), gamma-glutamyl transpeptidase (GGT) (103 U/L, normal range 5–60 U/L), and lactic dehydrogenase (LDH) (1692 U/L, normal range 15–210 U/L). Therefore, cachexia was diagnosed as her estimated Eastern Cooperative Oncology Group (ECOG) score was 3 due to her impaired nutrition and performance status, and best supportive care was administered initially.

**Figure 1:**
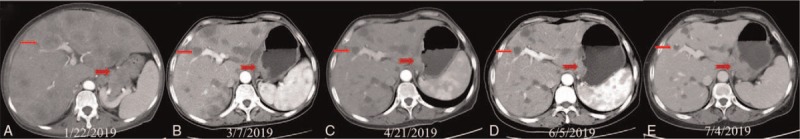
The CT images of the GEJ and liver metastasis during apatinib and S-1 treatment (The lesions were indicated by arrows). A. The GEJ tumor and markedly enlarged liver with diffused metastases were indicated on January 22, 2019. B. After 1 cycle of therapy, the hepatic metastases showed PR on March 7, 2019. C. The GEJ and liver masses were stable after 2 cycles of treatment on April 21, 2019. D. Three cycles later, the GEJ and hepatic lesions remained SD on June 5, 2019. E. Four cycles later, the hepatic lesions showed SD, but the GEJ tumor was noticeably thickened on July 4, 2019. CT = computed tomography, GEJ = gastroesophageal junction, PR = partial response, SD = stable disease.

**Figure 2:**
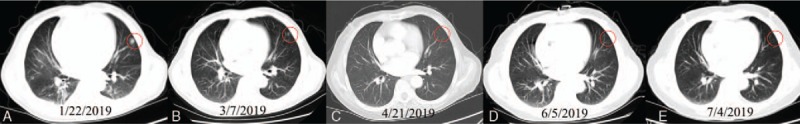
The CT images of the pulmonary nodule in left upper lobe. A. The lung nodule was indicated on January 22, 2019. B. After 1 cycle of treatment, the nodule showed PR on March 7, 2019. C. The nodule disappeared (CR) after 2 cycles of therapy on April 21, 2019. D. Recurrence of the nodule was excluded on June 5, 2019. E. Four cycles later, the tumor lesion was undetectable on July 4, 2019. CT = computed tomography.

On January 22, 2019, the gastric endoscopy and abdomen computed tomography (CT) scan revealed primary lesions in the lower esophageal region and in the cardiac end of the stomach. Further contrast-enhanced CT revealed irregularly thickened gastroesophageal junction (GEJ) wall, tumor-infiltrated adventitia, extensive distant metastasis (left supraclavicular and mediastinal lymph nodes), peritoneal effusion, and significantly enlarged liver with diffused metastases between the liver and stomach (Fig. 1). In addition, a pulmonary lesion located in the left upper lobe were also indicated (Fig. 2).

Endoscopic biopsy revealed the diagnosis of poorly-differentiated GEJ adenocarcinoma, and further immunohistochemistry stain of the specimen showed positive human epidermal growth factor receptor 2 (HER-2) and vascular endothelial growth factor (VEGF). Other metastasis was excluded using cranial magnetic resonance and whole-body bone emission CT. Then she was staged as cT3NxM1G3 (IVB) according to the 8th edition of tumour, node and metastasis (TNM) staging system for esophageal cancer.^[3]^

After a multidisciplinary evaluation, first-line palliative oral apatinib (425 mg, daily) plus S-1 (40 mg twice daily for 4 weeks, with a 2-week drug-free interval, as her body surface area was <1.5 m^2^) was administered for this patient. Informed consent was obtained from the patient prior to treatment. During the treatment, CT scans and serum CA125 were conducted irregularly for efficacy evaluation in accordance with the Response Evaluation Criteria in Solid Tumors (RECIST 1.1), meanwhile, the treatment-related side effects were recorded as National Cancer Institute Common Terminology Criteria for Adverse Events version 4.0.

**Figure 3:**
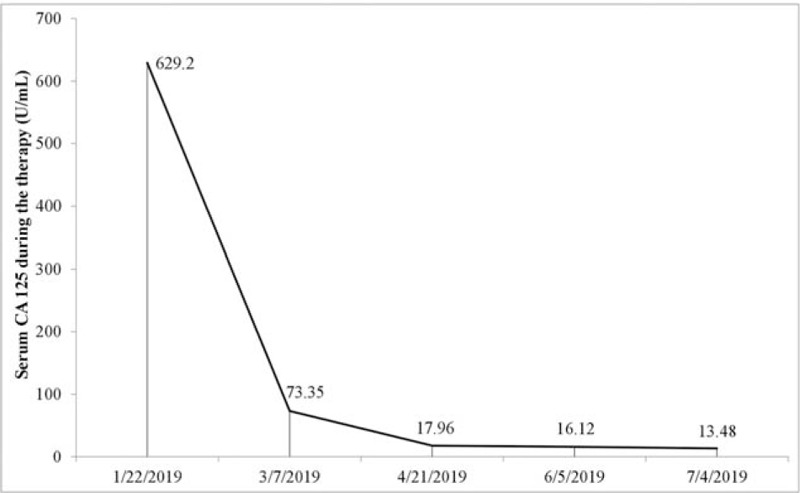
The serum CA125 was decreased gradually after S-1plus apatinib therapy.

Encouragingly, the GEJ tumor and the hepatic lesions showed stable disease (SD) and partial response (PR) respectively after 1 cycle of therapy using apatinib plus S-1, nevertheless, these tumors maintained SD after another 3 cycles of treatment without dosage reduction of the 2 agents (Fig. 1). In addition, the pulmonary lesion demonstrated a complete remission after 2 cycles of treatment (Fig. 2), although a pathological diagnosis was not obtained, because a CT-guided biopsy of this lesion was not performed with the aim to diminish unnecessary injury to the patient. Meanwhile, the serum CA125 was decreased steadily as shown in Fig. 3. Grade 2/3 toxicities including hypertension and hand-foot syndrome were tolerable after proper treatment. No grade 4 adverse event was observed in this case.

On July 4, 2019, the patient showed improved status as her ECOG score was 1. Although the hepatic lesions remained SD, the GEJ tumor was noticeably thickened (Fig. 1F). However, the patient refused docetaxel and oxaliplatin as second-line therapy and continued to take low-dose apatinib (250 mg, daily) and S-1 thereafter. This patient demonstrated a progression-free survival (PFS) of 5 months and an overall survival (OS) of 11 months up to November 12, 2019.

## 3 Discussion

The prognosis of advanced gastric and GEJ cancer patients remains dismal regardless of the current advances in anti-cancer treatment, underlining an urgent need of new therapeutic options, additionally, palliative chemo- or targeted-therapy might be considered for metastatic or recurrent disease.

S-1 that consists of tegafur, gimeracil, and oteracil potassium is a recommended first-line treatment for gastrointestinal cancers in Japan for advanced gastric cancer (GC).^[4]^ However, a retrospective study does not support the survival benefit of first-line S-1 plus cisplatin over S-1 monotherapy in advanced or recurrent GC patients aged ≥70 years.^[5]^ Paclitaxel plus S-1 shows a lower progressive disease rate as comparing to paclitaxel plus intravenous 5-fluorouracil.^[6]^ Additionally, it is reported that S-1-containing regimens could not improve the survival but increase the hematological toxicities in Asian patients as compared with 5-FU-containing regimens.^[7]^ As a consequence, a biweekly S-1 regimen might be considered in GC patients to diminish gastrointestinal and hematological toxicities.^[8]^

Tumor angiogenesis plays an important role in cancer metastasis, however, only very few approved agents lead to a clinical improvement in metastatic GC patients.^[9]^ Apatinib significantly improve the OS and PFS in metastatic gastric or GEJ cancer patients who are refractory to ≥2 lines of prior chemotherapy, and the most common grade 3/4 adverse events are hand-foot syndrome, proteinuria, and hypertension.^[10]^ A meta-analysis of randomized controlled trials reveals the significant efficacy and safety of VEGFR-2 inhibitors in metastatic gastric and GEJ cancer patients.^[11]^ Another network meta-analysis shows that apatinib, regorafenib, and rilotumumab improve the PFS and OS of advanced GC patients.^[12]^ Furthermore, apatinib can modulate the tumor immunosuppressive microenvironment, which is associated with the treatment resistance to programmed cell death protein 1 (PD-1) inhibitors.^[13]^ Further studies of anti-angiogenic therapy combined with chemotherapy or immune checkpoint inhibitors provide hope for GC patients. The addition of apatinib (500 mg/d) to chemotherapy could further improve the PFS of metastatic GEJ adenocarcinoma patients refractory to prior chemotherapy.^[14]^

It is noteworthy that, high-quality studies assessing the efficacy of apatinib plus S-1 in advanced gastric or esophageal cancer remain scarce. A meta-analysis of the reports published in Chinese language indicates that S-1 plus apatinib shows a favorable efficacy in GC patients as compared with S-1 alone,^[15]^ but it can be difficult to draw reliable conclusions due to the low quality of these included studies. The registered trials evaluating the efficacy of S-1 plus apatinib for gastric or gastroesophageal cancer are listed in Table 1, as better evidence are necessary before the incorporation of this regimen into practice guidelines.

**Table 1:**
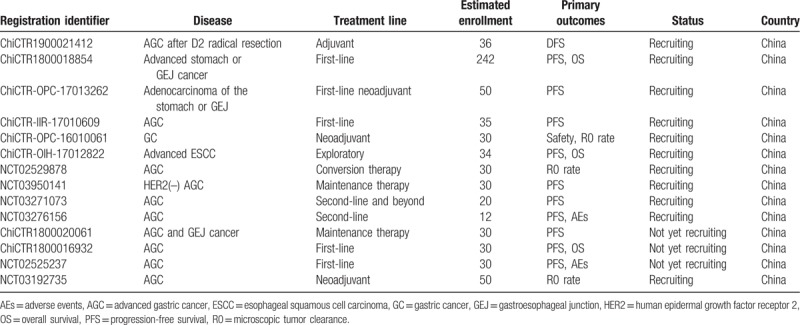
The registered trials evaluating the efficacy of S-1 plus apatinib for gastric or gastroesophageal cancer patients.

Anti-angiogenic strategy has major practical limitations such as inevitable resistance, and there are no definitive biomarkers for apatinib in GC.^[16]^ To the best of our knowledge, there is only one case report in English language, which shows that first-line apatinib plus S-1 in metastatic GC is effective based on peripheral circulating tumor cell monitoring.^[17]^ As for the presented case, favorable oncological outcome was achieved as she gained a 5-month PFS and 11 months of OS after the administration of concurrent apatinib and S-1, however, acquired resistance appeared after 4 cycles of treatment.

On the other hand, because of the heterogeneous nature of GC, it is not possible to evaluate a standard therapeutic approach. The addition of anti-VEGF agents to chemotherapy significantly improve the OS of advanced GC patients when compared with chemotherapy alone.^[18]^ But there is still no conclusive agreement on how to combine anti-angiogenic agents with other regimens to get prime effects. The most widely accepted first-line chemotherapy regimen for advanced GC is the combination of platinum and fluoropyrimidine, and trastuzumab could be considered if the tumor harbors positive human epidermal growth factor receptor type 2 (HER2).^[19,20]^ Furthermore, trastuzumab plus S-1 demonstrates promising activity in elderly HER2-positive, advanced GC patients,^[21]^ however, the optimal second- and third-line therapy is still a matter of debate. Ramucirumab plus taxane is the optimal second-line treatment, followed by taxane or irinotecan monotherapy as alternatives. Moreover, apatinib monotherapy is preferred in third-line setting,^[16,22–24]^ although apatinib shows modest antitumor activity.^[25]^

From this report, two questions arise: How to conquer acquired resistance of S-1 plus apatinib? What is optimal duration of S-1 plus apatinib as first-line palliative chemotherapy? To answer these questions, more researches are needed.

## 4 Conclusions

Oral S-1 plus apatinib might be a reasonable first-line palliative option for late-stage GEJ adenocarcinoma patients with compromised performance status, however, high-quality trials are warranted.

## Author contributions

**Conceptualization:** Chu Zhang, Dong Liu.

**Data curation:** Guang-Mao Yu.

**Funding acquisition:** Miao Zhang.

**Methodology:** Dong Liu.

**Resources:** Dong Liu.

**Writing – original draft:** Chu Zhang, Guang-Mao Yu.

**Writing – review & editing:** Chu Zhang, Miao Zhang, Dong Liu.

Chu Zhang orcid: 0000-0001-5860-8500.

Guang-Mao Yu orcid: 0000-0002-1737-9243.

Miao Zhang orcid: 0000-0001-7431-5986.

Dong Liu orcid: 0000-0003-2071-4548.
